# Demethylation and microRNA differential expression regulate plasma-induced improvement of chicken sperm quality

**DOI:** 10.1038/s41598-019-45087-1

**Published:** 2019-06-20

**Authors:** Jiao Jiao Zhang, Nisansala Chandimali, Nameun Kim, Tae Yoon Kang, Seong Bong Kim, Ji Su Kim, Xian Zhong Wang, Taeho Kwon, Dong Kee Jeong

**Affiliations:** 1 Chongqing Key Laboratory of Forage and Herbivore, College of Animal Science and Technology, Southwest University, Chongqing, 400715 P.R. China; 2 Laboratory of Animal Genetic Engineering and Stem Cell Biology, Department of Advanced Convergence Technology and Science, Jeju National University, Jeju, 63243 Republic of Korea; 3 Plasma Technology Research Center, National Fusion Research Institute, Gunsan-si, Jeollabuk-Do 54004 Republic of Korea; 4 Primate Resources Center, Korea Research Institute of Bioscience and Biotechnology, Jeongeup-si, Jeonbuk 56216 Republic of Korea

**Keywords:** Genetics, Developmental biology

## Abstract

The sperm quality is a vital economical requisite of poultry production. Our previous study found non-thermal dielectric barrier discharge plasma exposure on fertilized eggs could increase the chicken growth and the male reproduction. However, it is unclear how plasma treatment regulates the reproductive capacity in male chickens. In this study, we used the optimal plasma treatment condition (2.81 W for 2 min) which has been applied on 3.5-day-incubated fertilized eggs in the previous work and investigated the reproductive performance in male chickens aged at 20 and 40 weeks. The results showed that plasma exposure increased sperm count, motility, fertility rate, and fertilization period of male chickens. The sperm quality-promoting effect of plasma treatment was regulated by the significant improvements of adenosine triphosphate production and testosterone level, and by the modulation of reactive oxygen species balance and adenosine monophosphate-activated protein kinase and mammalian target of rapamycin pathway in the spermatozoa. Additionally, the plasma effect suggested that DNA demethylation and microRNA differential expression (a total number of 39 microRNAs were up-regulated whereas 53 microRNAs down-regulated in the testis) regulated the increases of adenosine triphosphate synthesis and testosterone level for promoting the chicken sperm quality. This finding might be beneficial to elevate the fertilization rate and embryo quality for the next generation in poultry breeding.

## Introduction

Fertility is a trait of major interest in the poultry industry because it determines the profitability of production. Chicken sperm quality influences the male fertility and the hatchability of fertilized eggs, which are the ultimate objective of poultry breeder management. Spermatozoa are highly specialized cells which require high adenosine triphosphate (ATP) level for providing the energy to ensure motility and fertilization potential^1^. Avian sperm shows specific features associated with the complex system of internal fertilization^2^. The intermediate piece of chicken sperm contains numerous mitochondria, which provide the energy for flagellum movement to reach the fertilization site in the infundibulum^3^. These biological features infer an important role of energetic metabolism in sperm to ensure the fertilizing ability of male chickens.

Non-thermal plasmas have the potential for a wide-range of biological applications in living cells and tissues^4^ because of no substantial gas heating. Our laboratory has established a non-thermal dielectric barrier discharge (DBD) plasma system generated in argon at atmospheric pressure for creating electrically safe plasma^5–9^. Our previous studies have suggested that appropriate non-thermal DBD plasma treatment conditions need to be optimized for the development of chicken embryos at stage Hamburger-Hamilton (HH) 20^7^. Primordial germ cells in chicken embryos at stage HH 20 have migrated and colonized into embryonic gonadal ridges after 3.5-day-incubation of fertilized eggs, at which stage external stimulation may affect the differentiation process of germ cells and the formation of gonads. Our previous work found 3.5-day-incubated fertilized eggs exposed to 2.81 W of plasma for 2 min exhibited significant improvements in the chicken growth within 3 months and in the sperm quality of 40-week-old male chickens^6^. Continually, we investigated the regulatory mechanism of non-thermal DBD plasma treatment on the sperm quality and whether plasma treatment can prolong the fertilization period of male chickens.

Gene expression is regulated by various factors, including DNA methylation, transcription factor, and microRNAs (miRNAs) pre- and post-transcription^10^. DNA methylation is a central epigenetic modification of gene expression and plays a crucial role in transcriptional regulation and genome transcription stability^11^. The methylation level within promoters or regulatory elements is negatively correlated with gene transcription in birds^12^ and demethylation generally correlates with the activation of gene expression^13^. Epigenetic modification in mature spermatozoa regulates the transcription of imprinted genes which determine sperm count and motility^14,15^ and early embryonic development^16^. Therefore, sperm DNA methylation are used to predicted male fertility and embryo quality during fertilization^17^. Genes that improve the development and differentiation are generally hypomethylated in the spermatozoa^16^, whereas hypermethylation results in spermatogenesis impairment and sperm quality defect^18^. Thus, these findings prompt that sperm quality may be influenced by DNA methylation level following the non-thermal DBD plasma exposure.

Besides DNA methylation is involved in the differential gene expression without sequence changes^19^, miRNAs also regulate gene expression and play important roles in cell proliferation, migration, and differentiation during animal development^20,21^. Increasing evidences support the notion that miRNAs are involved in the hypermethylation of sequences, which cumulatively contribute to epigenetic gene silencing^22^. DNA methylation on miRNAs indirectly affects the regulation of target genes, which results in silencing or over-expression in cases of hypo- or hyper-methylation of miRNAs, respectively^23^. Target gene expression is inversely correlated with methylation and the expression of corresponding miRNA^24^ at the pre- and post-transcriptional levels^25^. Thus, some candidate genes may be doubly regulated by DNA methylation and miRNAs with differential expression levels in the plasma-treated male chickens.

Numerous small pores in the palisades of chicken eggshell allow the potential diffusion of active charged and neutral particles in plasma, which generates reactive oxygen species (ROS) when exposed on the surface of cells or tissues^26^. Diffusion of plasma-produced ROS or accumulation of plasma-stimulated intracellular ROS^27,28^ regulate cell proliferation and differentiation^29,30^, and even sperm quality and physiology^5,31^. Low and physiological concentrations of ROS stimulate the sperm capacitation and acrosome reaction to ensure fertilization^31^. Whereas high concentrations of ROS lead to decreased sperm motility, viability, and fertilization capacity^32^. In this study, we attempt to determine whether plasma-produced ROS or stimulation of intracellular ROS mediate the chicken sperm quality.

Therefore, we sought to investigate how non-thermal DBD plasma treatment regulates the ROS homeostasis and energetic metabolism through cytosine methylation and miRNA differential expression for improving the chicken sperm quality. The investigation of mechanism will increase convincingness before the large-scale application of plasma technique in the field of livestock farming widely.

## Results

### Effects of plasma treatment on sperm quality and structure, testosterone level, and mitochondrial respiratory enzyme in the spermatozoa

**Figure 1:**
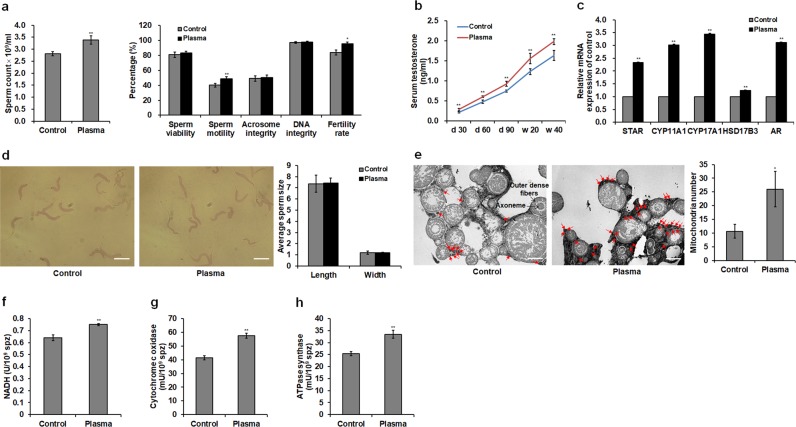
Sperm quality, testosterone level, sperm structure, and mitochondrial respiratory enzyme level in the spermatozoa. (**a**) Sperm count, viability, motility, integrities of acrosome and DNA, and fertility rate in 40-week-old male chickens. Sperm quality of 20-week-old male chickens are shown in Supplementary Fig. S1. (**b**) Testosterone levels in the serum of male chickens on days 30, 60, and 90, and weeks 20 and 40. For the sperm quality and testosterone level, data are presented as the mean ± standard deviation (SD) (n = 10) of three replicates; n represents an individual chicken. (**c**) Relative mRNA levels of testosterone biosynthesis genes [steroidogenic acute regulatory protein (*STAR*), cytochrome P450 family 11 subfamily A member 1 (*CYP11A1*), cytochrome P450 family 17 subfamily A member 1 (*CYP17A1*), and hydroxysteroid 17-beta dehydrogenase 3 (*HSD17B3*)] and androgen receptor (*AR*) gene in the testis of 40-week-old male chickens. (**d**) Representative sperm optical microstructure and average sperm size in 40-week-old male chickens. Scale bar: 5.0 μm. (**e**) Representative ultrastructure of transverse section of spermatozoa and mitochondria number. Mitochondria located around the outer dense fibers of the sperm midpiece are photographed. The red arrow shows the mitochondrion. Scale bar: 2.0 μm. (**f**) Nicotinamide adenine dinucleotide hydrogen (NADH) levels and activities of (**g**) cytochrome c oxidase and (**h**) ATPase synthase in the mitochondria of spermatozoa. For the mRNA level, mitochondrial number, and mitochondrial respiratory enzyme level, data are presented as the mean ± SD (n = 3) of three replicates; n represents an individual chicken. *p < 0.05 versus control; **p < 0.01 versus control, according to the one-way ANOVA with a least significant difference (LSD) test.

Plasma exposure at 2.81 W of discharge power for 2 min, which was applied as the optimal condition in respect of highest growth rate in chickens in our previous work^6^, showed a beneficial effect on the sperm quality of male chickens whose testis and sexual organs have been completely mature. Plasma treatment increased sperm count by 0.28-fold (*p* < 0.001) and motility by 0.16-fold (*p* < 0.001) in 20-week-old male chickens. The results obtained from the *in vivo* experiment showed that the fertility rate in hens inseminated with semen from plasma-treated male chickens aged at 20 weeks exhibited a 0.15-fold (*p* = 0.014) increase. However, sperm viability, integrity of acrosome and DNA non-significantly increased (see Supplementary Fig. S1). In order to study whether optimal plasma treatment can prolong the fertilization period of male chickens, we evaluated the sperm quality of male chickens aged at 40 weeks. The result showed that sperm count and motility and fertility rate in plasma-treated group were increased by 0.20- (*p* < 0.001), 0.22- (*p* < 0.001), and 0.13-fold (*p* = 0.018), respectively (Fig. 1a). In Fig. 1b, serum testosterone concentrations significantly increased in plasma-treated male chickens on days 30, 60, and 90, and at weeks 20 and 40, with increases of 0.27-fold (*p* < 0.001) in 20-week-old male chickens and 0.21-fold (*p* < 0.001) in 40-week-old male chickens exposed to plasma compared to those in controls. Plasma treatment also increased the mRNA expression of steroidogenic acute regulatory protein (*STAR*), cytochrome P450 family 11 subfamily A member 1 (*CYP11A1*), cytochrome P450 family 17 subfamily A member 1 (*CYP17A1*), hydroxysteroid 17-beta dehydrogenase 3 (*HSD17B3*), and androgen receptor (*AR*) in the testis of chickens aged at 40 weeks (Fig. 1c).

The optical microstructure of spermatozoa obtained from 40-week-old male chickens was normal and complete, and there was no significant difference in sperm morphology, as evidenced by the average sperm length and width between the plasma group and control group (*p* > 0.05; Fig. 1d); this indicated that the plasma treatment did not change the sperm morphology, and therefore ensured the normal fertilizing ability of spermatozoa. The number of mitochondria located around the outer dense fibers in the transverse section of the sperm midpiece in plasma-treated males was higher than in the control group, showing a 1.44-fold increase compared to the control group (*p* = 0.035; Fig. 1e). In addition, the plasma exposure increased the levels of nicotinamide adenine dinucleotide hydrogen (NADH) (Fig. 1f), cytochrome c oxidase (Fig. 1g), and ATPase synthase (Fig. 1h) by 0.17- (*p* = 0.004), 0.32- (*p* = 0.003), and 0.39-fold (*p* = 0.001) in spermatozoal mitochondria.

### Plasma treatment improves ATP level and regulates adenosine monophosphate-activated protein kinase (AMPK)-mammalian target of rapamycin (mTOR) pathway in the spermatozoa

**Figure 2:**
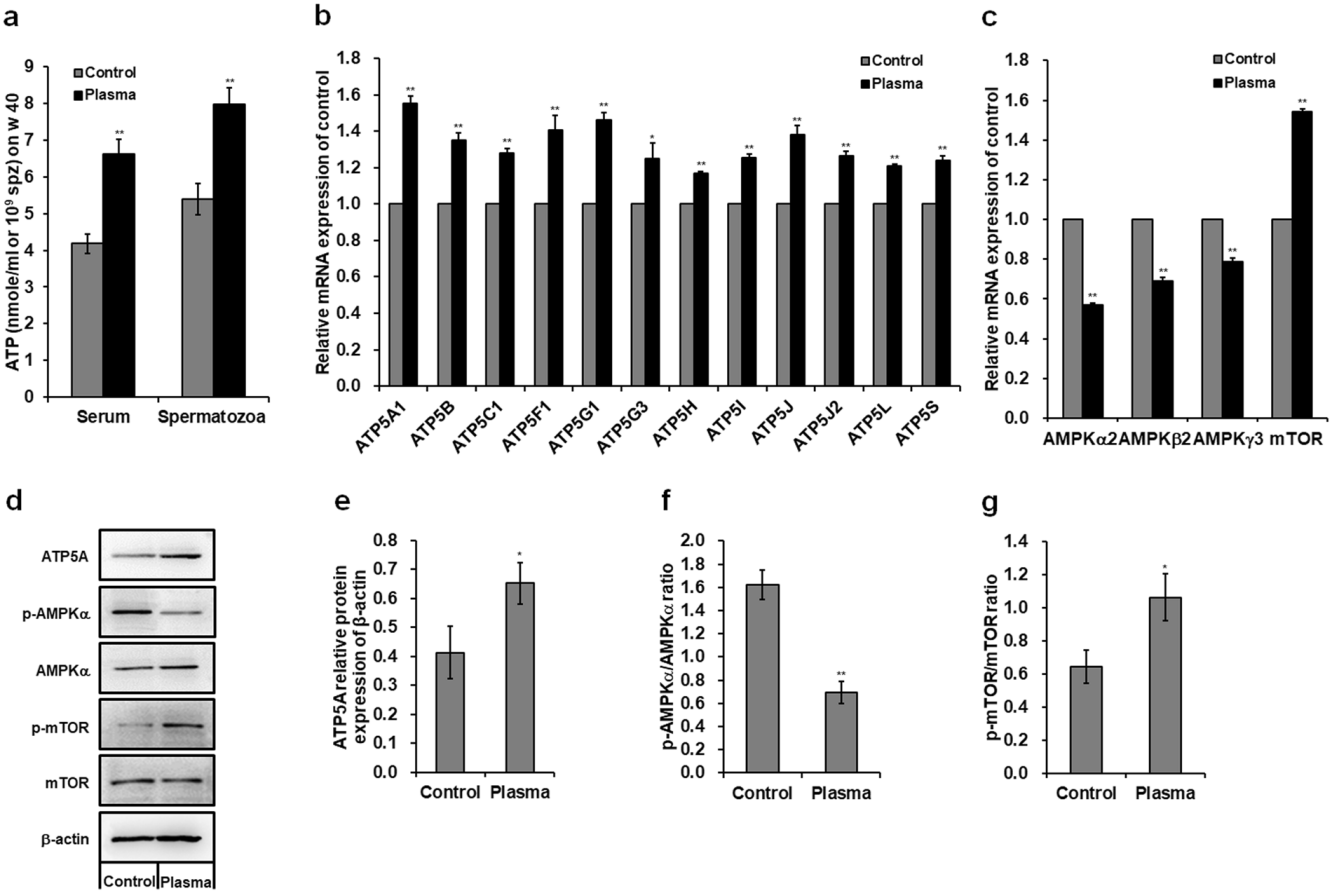
Adenosine triphosphate (ATP) level and adenosine monophosphate-activated protein kinase (AMPK)-mammalian target of rapamycin (mTOR) signaling pathway. (**a**) ATP concentrations in the serum and spermatozoa of 40-week-old male chickens. For the ATP concentration, data are presented as the mean ± SD (n = 10) of three replicates; n represents an individual chicken. Relative mRNA levels of (**b**) *ATP5* synthases, and (**c**) *AMPKα*2, *AMPKβ*2, *AMPKγ*3, and *mTOR* in the spermatozoa of 40-week-old male chickens. (**d**) Western blot analysis of protein bands in the spermatozoa. The grouping of gels/blots cropped from different gels. All bolts are visualized with 5 min exposure time. Uncropped immunoblot scans are shown in Supplementary Fig. S2. Relative protein levels of (**e**) ATP5A, (**f**) p-AMPKα/AMPKα, and (**g**) p-mTOR/mTOR. For the mRNA and protein level, data are presented as the mean ± SD (n = 3) of three replicates; n represents an individual chicken. *p < 0.05 versus control; **p < 0.01 versus control, according to the one-way ANOVA with a LSD test.

The plasma treatment significantly increased the ATP levels in the serum and spermatozoa of 40-week-old male chickens (Fig. 2a), with a 0.59- (*p* < 0.001) and 0.48-fold (*p* < 0.001) increase in the serum and spermatozoa, respectively, compared to those in the control group. Plasma treatment also significantly up-regulated the mRNA expression of *ATP5* synthases in the spermatozoa (Fig. 2b), which catalyze ATP synthesis. mRNA level of *ATP5A1* which encodes a subunit of mitochondrial ATP5 synthases exhibited a maximum increase of 0.55-fold (*p* < 0.001; Fig. 2b) among those *ATP5* synthases compared to the control group. Moreover, plasma exposure increased ATP5A protein expression in the spermatozoa, with an increase of 0.58-fold (*p* < 0.001) compared to that in the control (Fig. 2d,e). Uncropped immunoblots are presented in Supplementary Fig. S2. In Fig. 2c, plasma treatment significantly up-regulated *mTOR* mRNA expression but down-regulated *AMPK* mRNA expression in the spermatozoa compared to those in the control groups. In addition, plasma exposure increased mTOR phosphorylation by 0.65-fold (*p* = 0.001; Fig. 2d,g), whereas decreased AMPKα phosphorylation by 0.57-fold (*p* < 0.001; Fig. 2d,f) in the spermatozoa compared to those in the controls.

### Plasma treatment regulates DNA methylation level

**Figure 3:**
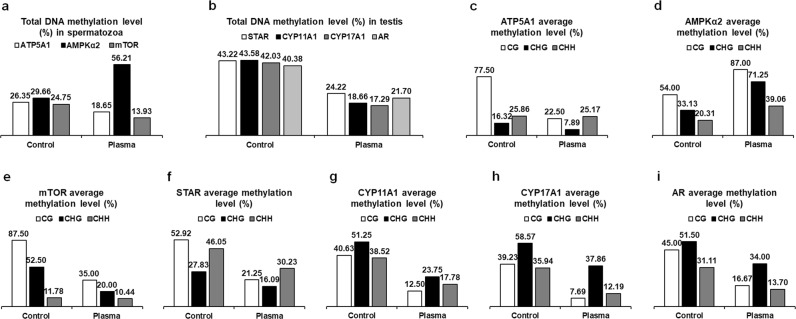
DNA methylation levels in the spermatozoa and testis of 40-week-old male chickens. Total DNA methylation levels in the sequenced regions of (**a**) *ATP5A1*, *AMPKα*2, and *mTOR* in the spermatozoa and (**b**) *STAR*, *CYP11A1*, *CYP17A1*, and *AR* in the testis. Total methylation ratios were calculated by dividing the number of non-converted (methylated) cytosines by the total number of cytosines within the sequenced region; values were expressed as percentages (%). Average methylation levels for CG, CHG, and CHH in the sequenced regions of (**c**) *ATP5A1*, (**d**) *AMPKα*2, and (**e**) *mTOR* in the spermatozoa, (**f**) *STAR*, (**g**) *CYP11A1*, (**h**) *CYP17A1*, and (**i**) *AR* in the testis. Average methylation levels were expressed as percentage (%) per site for each of the three types of cytosines (CG, CHG, and CHH), and were calculated by dividing the number of non-converted cytosines by the total number of cytosines of each type. The cytosine methylation analysis results see Supplementary Fig. S3. An independent replicate on DNA methylation levels in the spermatozoa and testis of 40-week-old male chickens see Supplementary Fig. S5.

Bisulfite sequencing results of *ATP5A1*, *AMPKα*2, *mTOR*, *STAR*, *CYP11A1*, *CYP17A1*, and *AR* were reported in Supplementary Fig. S3, showing the exact location, length of sequenced region, type, and extent of methylation. Compared to the control group, total methylation levels of three types of sites [cytosine guanine (CG), CHG, and CHH, where H equals adenosine, thymine, or cytosine] in the sequenced regions of *ATP5A1* and *mTOR* were decreased by 0.08- and 0.11-fold, respectively, in the spermatozoa of plasma-treated males aged at 40 weeks. However, plasma treatment increased total methylation level in the sequenced region of *AMPKα*2 by 0.27-fold in the spermatozoa (Fig. 3a). In addition, total methylation levels in the sequenced region of *STAR*, *CYP11A1*, *CYP17A1*, and *AR* were decreased by 0.19-, 0.25-, 0.25-, and 0.19-fold, respectively, in the testis of plasma-treated male chickens compared to those in the controls (Fig. 3b). Furthermore, the average methylation percentage of each type of sites showed that the variation amplitude of average methylation levels of those genes for the CG type was greater than CHG and CHH following the plasma exposure (Fig. 3c–i).

### Plasma treatment induces differentially expressed miRNAs in the testis

**Figure 4:**
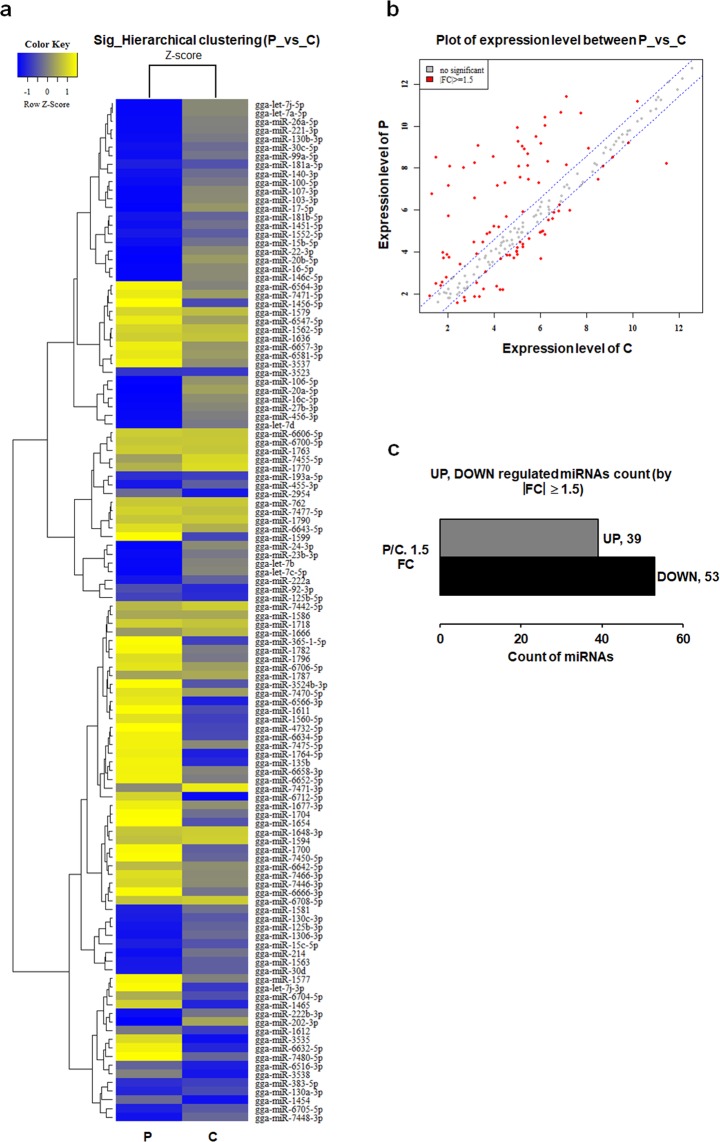
MicroRNA (miRNA) expression levels in 40-week-old male chicken testis. (**a**) Hierarchical cluster analysis of miRNA expression levels. Heat map representation of miRNAs that differed significantly between plasma treatment (P) and control (C) groups. Rows represent transcriptional units. miRNAs that share a similar trend of ascending or descending property are clustered. The yellow represents the maximum Z-score; the blue represents the minimum Z-score. (**b**) Scatter plot of miRNA expression levels. Red dots represent significant miRNAs; grey dots represent no significant miRNAs. (**c**) Bar plot of significant miRNAs. FC, fold change. miRNA with |FC| ≥ 1.5 and a p-value < 0.05 is significantly up- or down-regulated. The detailed information of significant miRNAs see Supplementary Table S1.

Figure 4a showed the hierarchical cluster analysis image of miRNA expression in the testis of 40-week-old male chickens with 92 miRNAs (accounting for 36.95% of the total number of 249 miRNAs examined) with significant up- and down-regulation. Based on hierarchical clustering analyses, the expression patterns of these 92 miRNAs were subjected to regulating tendency selection processes. A scatter plot of differentially expressed miRNAs between the plasma-treated chickens and controls was shown in Fig. 4b. Thirty-nine miRNAs with significant up-regulation and 53 miRNAs with significant down-regulation were found in the plasma-treated group (Fig. 4c). The detailed information of these differentially expressed miRNAs between plasma-treated and control groups were shown in Supplementary Table S1. The putative targets of significantly expressed miRNAs were predicted by miRDB (http://www.mirdb.org/). Target genes of 21 miRNAs were associated with changes in mRNA expression levels following the plasma treatment (Table 1). *AMPK* was a putative target gene of 2 up-regulated miRNAs (miR-7450-5p and miR-2954). Among the 19 down-regulated miRNAs, *mTOR* was a putative target gene of miR-99a-5p and miR-100-5p; *STAR* was a putative target gene of miR-106-5p (Table 1). The three target genes of above five miRNAs were measured with changes in the DNA methylation level and mRNA expression. The putative targets of remaining 16 down-regulated miRNAs were found with changes in the mRNA expression following the plasma treatment; of these, putative targets of 10 down-regulated miRNAs were *ATP5* synthases (Table 1).

**Table 1:** Key microRNAs (miRNAs) with differential expression in the testis of 40-week-old male chickens.

MiRNA name	Sequence	Sequence cordinate	P/C. FC	Associated target genes
gga-miR-7450-5p	UCUGUUCUUAAGGAGGCUGAGGC	chr1:54894245-54894267 (+)	1.81568	*AMPK*
gga-miR-2954	CAUCCCCAUUCCACUCCUAGCA	chrZ:70557207-70557228 (+)	1.543874	*AMPK*
gga-miR-130a-3p	CAGUGCAAUAUUAAAAGGGCAU	chr15:399369-399390 (−)	−1.606146	*PRDX3, HSD17B3*
gga-miR-222a	AGCUACAUCUGGCUACUGGGUCUC	chr1:109977233-109977256 (+)	−1.949927	*ATP5L*
gga-miR-30d	UGUAAACAUCCCCGACUGGAAG	chr2:142229538-142229559 (−)	−2.298415	*ATP5G1*
gga-miR-181b-5p	AACAUUCAUUGCUGUCGGUGGG	chr17:9498558-9498579 (+) // chr8:1986890-1986911 (+)	−3.837102	*PRDX3*
gga-miR-15b-5p	UAGCAGCACAUCAUGGUUUGCA	chr9:21653636-21653657 (−)	−7.086145	*ATP5C1, ATP5L, ATP5S*
gga-miR-23b-3p	AUCACAUUGCCAGGGAUUACC	chrZ:41508087-41508107 (+)	−7.314428	*ATP5G1*
gga-miR-99a-5p	AACCCGUAGAUCCGAUCUUGUG	chr1:98347485-98347506 (+)	−7.925797	*MTOR*
gga-miR-100-5p	AACCCGUAGAUCCGAACUUGUG	chr24:3330849-3330870 (+)	−8.741549	*MTOR*
gga-miR-130b-3p	CAGUGCAAUAAUGAAAGGGCGU	chr15:390547-390568 (−)	−9.207497	*PRDX1, HSD17B3*
gga-miR-456-3p	CAGGCUGGUUAGAUGGUUGUCA	chr3:30789084-30789105 (−)	−10.561713	*ATP5S*
gga-miR-221-3p	AGCUACAUUGUCUGCUGGGUUUC	chr1:109977744-109977766 (+)	−11.892606	*ATP5L*
gga-miR-107-3p	AGCAGCAUUGUACAGGGCUAUCA	chr6:18945422-18945444 (−)	−12.796044	*ATP5S*
gga-let-7b	UGAGGUAGUAGGUUGUGUGGUU	chr1:71371984-71372005 (+)	−13.241843	*PRDX1, PRDX4*
gga-miR-26a-5p	UUCAAGUAAUCCAGGAUAGGC	chr2:4537775-4537795 (+)	−13.680907	*PRDX1*
gga-miR-103-3p	AGCAGCAUUGUACAGGGCUAUGA	chr13:4060451-4060473 (+)// chr4:88047793-88047815 (−)	−14.044584	*ATP5S*
gga-miR-24-3p	UGGCUCAGUUCAGCAGGAACAG	chrZ:41508848-41508869 (+)	−19.129192	*ATP5L*
gga-miR-16-5p	UAGCAGCACGUAAAUAUUGGUG	chr1:168694597-168694618 (−)// chr9:21653466-21653487 (−)	−33.39268	*ATP5C1*
gga-miR-27b-3p	UUCACAGUGGCUAAGUUCUGC	chrZ:41508332-41508352 (+)	−42.958092	*PRDX3*
gga-miR-106-5p	AAAAGUGCUUACAGUGCAGGUA	chr4:3946676-3946697 (−)	−63.056273	*STAR*

P, plasma treatment; C, control; FC, fold change. miRNA with |FC| ≥ 1.5 and a p-value < 0.05 is significantly up- or down-regulated. All differentially expressed miRNAs following the plasma treatment are shown in Supplementary Table S1.

### Gene ontology (GO) biological process enrichment and kyoto encyclopedia of genes and genomes (KEGG) pathway

To further understand the physiological functions and biological processes involved in target genes of differentially expressed miRNAs between the plasma-treated chickens and controls, we conducted a GO biological process enrichment and KEGG analysis. The highlighted counts of target genes of differentially expressed miRNAs were implicated in the regulation of transcription in the GO analysis (see Supplementary Table S2) and MAPK signaling pathway, which is involved in a series of protein kinase cascades and regulates the activities of several transcription factors to control the cellular processes, in the KEGG analysis (see Supplementary Table S3).

### Plasma treatment negatively regulates ROS and malondialdehyde (MDA) levels while positively regulates antioxidant enzyme levels in the serum and spermatozoa

**Figure 5:**
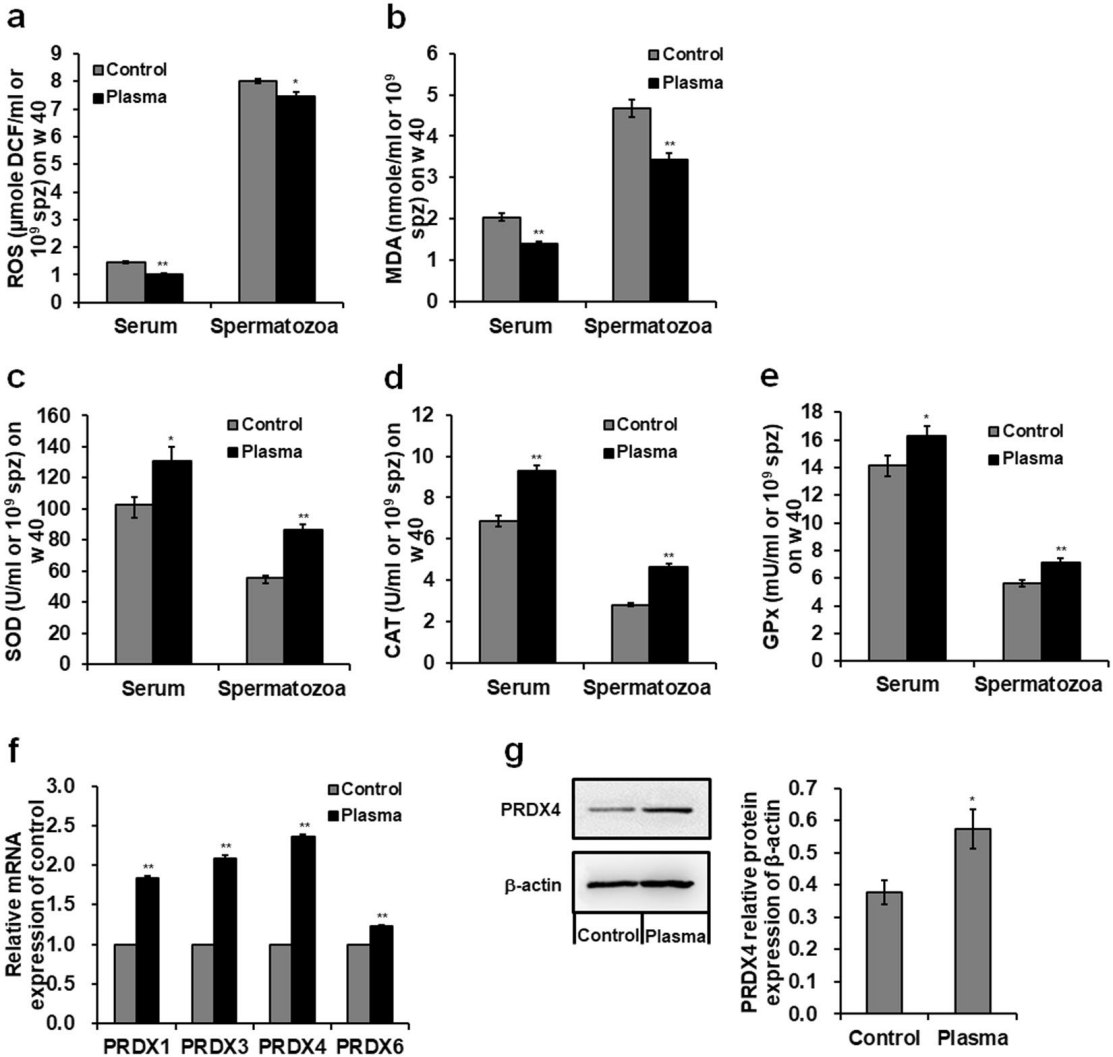
Reactive oxygen species (ROS), malondialdehyde (MDA), and antioxidant enzyme levels. Concentrations of (**a**) ROS and (**b**) MDA, and activities of antioxidant enzyme (**c**) superoxide dismutase (SOD), (**d**) catalase (CAT), and (**e**) glutathione peroxidase (GPx) in the serum and spermatozoa of 40-week-old male chickens. (**f**) Relative mRNA levels of peroxiredoxin (*PRDX*) 1, *PRDX3*, *PRDX4*, and *PRDX6* in the spermatozoa of 40-week-old male chickens. (**g**) Western blot analysis of protein bands and PRDX4 relative protein level in the spermatozoa. The grouping of gels/blots cropped from different gels. All bolts are visualized with 5 min exposure time. Uncropped immunoblot scans are shown in Supplementary Fig. S2. Data are presented as the mean ± SD (n = 3) of three replicates; n represents an individual chicken. *p < 0.05 versus control; **p < 0.01 versus control, according to the one-way ANOVA with a LSD test.

The plasma treatment significantly decreased ROS and MDA levels but increased the concentrations of superoxide dismutase (SOD), catalase (CAT), and glutathione peroxidase (GPx) in the serum and spermatozoa of 40-week-old male chickens (Fig. 5a–e). Specifically, plasma-treated male chickens showed a 0.29- (p < 0.001) and 0.07-fold (*p* = 0.016) decrease in ROS (Fig. 5a); a 0.31- (*p* = 0.001) and 0.27-fold (*p* = 0.003) decrease in MDA (Fig. 5b), whereas, a 0.27- (*p* = 0.014) and 0.56-fold (*p* < 0.001) increase in SOD activity (Fig. 5c); a 0.36- (*p* = 0.001) and 0.64-fold (*p* < 0.001) increase in CAT (Fig. 5d); and a 0.15- (*p* = 0.049) and 0.27-fold (*p* = 0.006) increase in GPx (Fig. 5e) in the serum and spermatozoa, respectively, compared to those in the control group. In addition, plasma treatment significantly up-regulated peroxiredoxins (*PRDXs*) mRNA expression in the spermatozoa (Fig. 5f). *PRDX4* mRNA level showed a maximum increase of 1.36-fold (*p* < 0.001; Fig. 5f) among *PRDXs* compared to the control group. Moreover, plasma exposure increased PRDX4 protein expression in the spermatozoa, with an increase of 0.51-fold (*p* < 0.001) compared to that in the control (Fig. 5g).

## Discussion

As an innovative technology in biological applications, non-thermal DBD plasma has recently been developed for application in the treatment of wounds, cancers, dental decays, and dermatological indications, and enhancement in the cell transfection efficiency, cell proliferation, and tissue regeneration^33^. Our recent study suggested the optimized condition of non-thermal plasma treatment promoted the development of chicken embryos at stage HH 20^7^ and the chicken growth rate after hatching, especially higher growth characteristics in male chickens, and the improvement in sperm quality of 40-week-old male chickens^6^. However, there were no significant effects on the female reproductive performance following the plasma treatment^6^. Therefore, we focused on revealing the molecular mechanism how non-thermal DBD plasma regulates the sperm quality and whether plasma treatment can prolong the fertilization period of male chickens in this study. The results showed that plasma treatment (2.81 W for 2 min) on 3.5-day-incubated fertilized eggs improved sperm count, motility, and fertility rate of male chickens aged at 20 and 40 weeks. Although the gonads in chicken embryos are not yet formed after 3.5-day-incubation, primordial germ cells have migrated and colonized into embryonic gonadal ridges. Therefore, plasma treatment may affect the differentiation process of germ cells and the formation of gonads. Although sperm quantity and quality decrease as the age of male chickens increase, the values of sperm count, motility, and fertility rate in plasma-treated male chickens aged at 40 weeks were higher than those in controls, which suggested that optimal plasma treatment delayed the decline of sperm quality and prolonged the fertilization period of male chickens.

In the present study, we found that optimized non-thermal DBD plasma treatment increased sperm count, motility, and fertility rate but sperm morphology, viability, integrity of acrosome and DNA non-significantly increased, inferring that non-thermal DBD plasma treatment promoted the spermatogenesis and sperm motility and fertility, which may be regulated by the increase of ATP production because ATP plays a central role in spermatogenesis and sperm motility and fertilizing ability^1,34,35^. We also found that plasma treatment increased ATP levels in the serum and spermatozoa. High levels of ATP inactivate AMPK, which contributes to activate mTOR signaling pathway^36^, resulting in improvements of intracellular energy metabolism^3^ for sperm motility^37,38^ and testicular somatic cell proliferation^39^ which enhances spermatogenesis. In this study, our results revealed that plasma treatment decreased *AMPK* mRNA expression and its phosphorylation level, and increased *mTOR* mRNA expression and its phosphorylation level in spermatozoa. Therefore, plasma treatment promoted spermatogenesis and sperm motility and fertility via increasing ATP production and regulating the AMPK-mTOR pathway.

To explain how plasma treatment regulates the ATP production, we investigated the changes of mitochondria numbers, mitochondrial respiratory enzyme activity, and expressions of *ATP5* synthases in the spermatozoa. Our results showed plasma exposure enhanced mitochondria numbers and mitochondrial respiratory enzyme activity which contribute to increase the ATP production^40–42^ and up-regulated the *ATP5* synthases subunit mRNA expression and ATP5A protein which catalyze ATP synthesis in the spermatozoa. In addition, testosterone plays an important role in regulating ATP synthesis for the stimulation of energy metabolism^43^ and spermatogenesis^44^ in male animals. We found that plasma treatment increased serum testosterone concentration by up-regulating mRNA expressions of testosterone biosynthesis genes (*STAR*, *CYP11A1*, *CYP17A1*, and *HSD17B3*) in the testis. Therefore, plasma treatment enhanced ATP production by increasing mitochondria numbers and mitochondrial respiratory enzyme activity, and modulating *ATP5* synthases and testosterone biosynthesis.

To explore the molecular mechanism of plasma-regulated engergy metabolism in male reproductive system, we studied the changes of methylation levels of *ATP5* synthases, *AMPK*, and *mTOR* in the spermatozoa, and testosterone biosynthesis genes in the testis. We found that plasma-treated male chickens increased sperm DNA demethylation of *ATP5A1* and *mTOR* whereas decreased sperm DNA demethylation of *AMPK*. DNA methylation within promoters or regulatory elements is negatively correlated with gene transcription in birds^12^ and demethylation generally correlates with the activation of gene expression^13^. Therefore, mRNA expressions of *ATP5A1* and *mTOR* were up-regulated but *AMPK* mRNA expression was down-regulated following the plasma treatment. Sperm DNA methylation level is significantly associated with sperm count and motility but is not related to sperm vitality or morphology^15^; this may support the results in our study that plasma-treated male chickens were found with increased sperm count, motility and fertility rate, whereas no significant changes in the viability, integrity of acrosome and DNA, and morphology of spermatozoa. Moreover, DNA demethylation levels of testosterone biosynthesis genes (*STAR*, *CYP11A1*, and *CYP17A1*) and *AR* gene in the testis were increased in plasma-treated males. Therefore, mRNA expressions of testosterone biosynthesis genes and *AR* gene were up-regulated, resulting in a stimulated transcription of testosterone responsive genes and significant increase of serum testosterone, which contributes to stimulate energy metabolism^43^ and spermatogenesis^44^ for promoting the sperm count, motility, and fertility rate. Global sperm DNA hypermethylation level was revealed in the spermatozoa of infertile patients^18^. Hypomethylation level within developmentally important promoters was detected in the spermatozoa of fertile donors and subfertile men^45^. However, sperm DNA methylation patterns differ significantly and consistently for infertile, fertile, and normozoospermic men. In addition, DNA methylation patterns may be predictive of fertilization rate and embryo quality^17^. Because DNA methylation in spermatogenesis has important implications for stable transmission of epigenetic information to the offspring^46,47^, which contributes to early embryonic development, fetal growth, and post-natal behavior in the next generation^16,48^, there is a probability that the offspring of plasma-treated male chickens inherit those genes expression changes with higher fertilization rate and embryo quality in poultry breeding.

miRNAs sharing interacting target genes whose expression is inversely correlated with methylation level^24^ regulate the cellular process and metabolic pathway. Thirty-nine miRNAs up-regulated and 53 miRNAs down-regulated were found in plasma-treated chicken testis. Twenty-one miRNAs expression exhibited inverse changes with mRNA expression levels of their target genes because miRNAs induce mRNA degradation and translational inhibition^20^. He *et al*. provided clarification on how DNA methylation and miRNAs work together to regulate gene expression and explained the suppression or activation caused by DNA methylation and miRNA silencing on genes at the pre- and post-transcriptional levels^25^. In our study, 10 down-regulated miRNAs were found with up-regulation of mRNA expression of *ATP5* synthases. Three down-regulated miRNAs whose target genes had hypomethylation levels caused an up-regulation of mRNA expression of *mTOR* and *STAR*. Two up-regulated miRNAs whose target genes had hypermethylation levels caused a down-regulation of *AMPK* mRNA expression. This network illustrates a potential regulatory mechanism of DNA methylation and miRNAs on *ATP5* synthases, AMPK-mTOR pathway, and testosterone biosynthesis in the reproductive system of plasma-treated male chickens. However, validated experimental studies on this network mechanism and correlation between miRNAs and DNA methylation are worthy of further study.

Although ATP is necessary to support the spermatogenesis and sperm motility and fertilizing ability, ATP production generally accompanies the generation of ROS. The ROS homeostasis in avian semen is essential for sperm quality and fertilizing ability^31,32,35^. Non-thermal DBD plasma treatment-produced ROS and plasma-stimulated intracellular ROS generation^27,28^ regulate sperm quality and physiology^5,31^. Mitochondrial respiratory chain produces cellular ROS^49^ and influences the physiological levels of ROS in the plasma-induced oxidative stress^50^. ROS scavenging can be regulated by the catabolism in antioxidant enzymes^28,51,52^. Our study found plasma treatment controlled intracellular ROS relatively low and significantly reduced MDA activity, which were mediated by up-regulating antioxidant enzyme levels of SOD, CAT, and GPx and *PRDX* mRNA expression and PRDX4 protein level in the spermatozoa. These findings indicated that the plasma treatment regulates ROS homeostasis for ensuring the sperm quality and fertilizing ability in male chickens through up-regulating the antioxidant enzyme activity and influencing the mitochondrial respiratory chain.

In conclusion, non-thermal DBD plasma treatment of fertilized eggs before hatching resulted in the increase of sperm quality and the prolongation of fertilization period of male chickens. The sperm quality-improving effect was regulated by the promotion of energy metabolism via increasing the ATP production and modulating the AMPK-mTOR pathway in the spermatozoa. The plasma effect suggested that the DNA demethylation level and miRNA differential expression regulated the increases of ATP synthesis and testosterone level for promoting the chicken sperm quality. Additionally, the plasma treatment regulated ROS homeostasis for ensuring the sperm quality and fertilizing ability via up-regulating the antioxidant enzyme activity. The non-thermal DBD plasma treatment provides a potentially viable and safe strategy for improving the male reproductive capacity in chickens; this finding might be beneficial to obtain higher fertilization rate and embryo quality for the next generation in the poultry breeding.

## Materials and Methods

### Animals and plasma treatment

Artificial insemination was performed with semen obtained from the rooster (*Korean native chicken*, broiler). Fertilized eggs (70 eggs for each group) obtained from hens (*Hyline brown chicken*, layer; raised at a chicken farm in Jeju National University, Jeju, Republic of Korea) were incubated at 37.5 °C with a 45–65% relative humidity and rotated 90° every 2 h. Three point five-day-incubated eggs were kept in the plasma reactor, exposing to 2.81 W of discharge power for 2 min following our previously described method^6,7^. Plasma-treated fertilized eggs were incubated for 21 days and hatched out with a hatchability of approximately 80%. Chickens were housed in individual cages under the same environment conditions and given free access to equal amount of water and basic feed until the age of 40 weeks old. Commercial crumbles (containing 18.00% crude protein, 2.50% crude fat, 7.00% crude fiber, 0.85% lysine, 0.25% methionine, 1.00% calcium, 0.70% phosphorus, and 0.50% salt) were used for feeding chicks aged at day 1 to 8 weeks. Commercial pellet feeds (containing 17.00% crude protein, 2.50% crude fat, 8.00% crude fiber, 0.40% lysine, 0.25% methionine, 1.00% calcium, 0.40% phosphorus, and 0.50% salt) were used for feeding adolescent chickens aged at 8 to 40 weeks. There were no differences in feed intake for each group. Animal handling protocols were approved by the Institutional Committee for Ethics in Animal Experiments of Jeju National University (approval number: 2016-0022) and all experiments were performed in accordance with the institution guidelines.

### Serum testosterone, ATP, ROS, MDA, and antioxidant enzyme analyses

The serum samples of 10 male chickens on days 30, 60, 90, and at weeks 20 and 40 were collected and centrifuged for 20 min at 1,000 × g. Serum testosterone level was detected using the chicken testosterone ELISA kit (CUSABIO, Wuhan, Hubei, China) according to the manufacturer’s instructions. Semen of 10 male chickens at weeks 20 and 40 was centrifuged at 600 × g for 10 min. The pellet of spermatozoa was washed with 0.9% sodium chloride solution, centrifuged again, and suspended in PBS to achieve a final concentration of 1 × 10^9^ spermatozoa/ml. The serum and spermatozoa obtained from 40-week-old male chickens were analyzed for ATP, SOD, CAT, GPx, ROS, and MDA concentrations following our previously described method^5–7^.

### Sperm quality evaluation, transmission electron microscopy, and mitochondrial respiratory enzyme analyses

Ten male chickens in each group were randomly used for sperm quality evaluation. Sperm morphology, count, vitality, motility, and integrity of acrosome and DNA were evaluated as our previously described method^5^. In the experiment of fertility rate, 10 male chickens in the control group and plasma-treated group were used for the collection of semen respectively. Artificial insemination was performed once every two days and three times in total on 100 healthy hens (*Hyline brown chicken*, layer) in each group with 10 hens inseminated with semen from each male chicken. Two hundred of fertilized eggs in each group were obtained after 2-day collection. The fertility rate were evaluated as our previously described method^6^. Semen samples were fixed in a mixture of 2.5% glutaraldehyde in PBS (0.05 mol/l, pH 7.4) overnight at 4 °C. Spermatozoa were subsequently post-fixed in 1% osmium tetroxide for 1 h, dehydrated in acetone and embedded in Epon Araldite. Ultrathin sections (50–70 nm) were cut and mounted on copper grids, stained with uranyl acetate and lead citrate, and then photographed using the MegaView Soft Imaging System (Olympus, Tokyo, Japan) at 120 kV. The mitochondria of spermatozoa were isolated and purified using Qproteome Mitochondria Isolation Kit (QIAGEN, Valencia, CA, USA) according to the manufacturer’s protocol. The mitochondrial respiratory enzyme concentrations were detected as our previously described method^5^.

### RT-PCR analysis

Chicken sperm total RNA was isolated and purified using the RNAzol method described by Shafeeque *et al*.^53^. Sperm RNA quality and concentration were assessed using a NanoDrop 2000 (Thermo Fisher Scientific, Waltham, MA, USA). The absorbance ratios at 260/280 and concentrations of chicken total sperm RNA in the control and plasma treatment group were shown in Supplementary Table S4. cDNA synthesis was performed using TOPscript^TM^ RT DryMIX (dT18) (Enzynomics, Daejeon, Republic of Korea). RT-PCR analysis was performed using Prime Taq Premix (2×) (GENETBIO, Yuseong-gu, Daejeon, Republic of Korea), and EvaGreen Dye (Biotium, Hayward, CA, USA) according to the manufacturer’s instructions. Primer sequences for RT-PCR are shown in Supplementary Table S5. mRNA relative expression levels were normalized to the housekeeping gene (*β-actin*) and calculated using the 2^−ΔΔCT^ method.

### Methylation sequencing

Genomic DNA was isolated and purified from chicken spermatozoa and testis using an AllPrep DNA/RNA Micro Kit (QIAGEN). Sodium bisulfite conversion bisulfite-sequencing PCR (BSP) were performed using the EpiTech Bisulfite Kit (QIAGEN). The original sequence and the bisulfite converted sequence of sequenced region were shown in Figure S4. BSP primers were designed using MethPrimer (http://www.urogene.org/methprimer/) (see Supplementary Table S6). BSP products were purified, ligated, and transformed using the pGEM-T Easy Vector system I (Promega, Madison, WI, USA). Plasmids extracted from ten positive clones which contain the target DNA were confirmed and sequenced as previously described method^5,8^. The cytosine methylation was analyzed using CyMATE software. The total methylation ratio and average methylation levels for CG, CHG and CHH were calculated as previously described method^5,8^.

### Western blotting

Sperm total protein was extracted as the method described by Labas *et al*.^54^. The protein concentration was measured using the Bicinchoninic Acid Protein Assay Kit (Sigma-Aldrich, St. Louis, MO, USA) and adjusted to equal protein concentration. Western blotting was performed as our previously described method^5,7,9,55^. The information of antibody dilutions was shown in Supplementary Table S7. Band intensity was quantified using the ImageJ software (National Institutes of Health, Bethesda, MA, USA). The densitometric value of each p-AMPKα, AMPKα, p-mTOR, and mTOR band was normalized to the β-actin before calculating the p-AMPKα/AMPKα and p-mTOR/mTOR ratios. The densitometric values of the ATP5A and PRDX4 bands were also normalized to the relevant β-actin.

### miRNA array

Total RNA was isolated and purified from chicken testis using the TRIzol reagent (Invitrogen, Thermo Fisher Scientific). Total RNA was labeled using the FlashTag Biotin RNA Labeling Kit (Genisphere, Hatfield, PA, USA). The labeled RNA was quantified, fractionated, and hybridized using GeneChip Hybridization Oven 645 (Affymetrix, Santa Clara, CA, USA) and the Affymetrix miRNA microarray. The labeled RNA was heated to 99 °C for 5 min and then to 45 °C for 5 min. RNA-array hybridization was performed with agitation at 60 rotations per minute at 48 °C for 16 h. The chips were stained using a Genechip Fluidics Station 450 (Affymetrix) and scanned with the Genechip Scanner 3000 7 G (Affymetrix). Signal values were determined using the Affymetrix GeneChip Command Console software. The results were analyzed using Affymetrix Expression Console Software. Hierarchical cluster was analyzed using the complete linkage and Euclidean distance. All statistical tests and visualization of differentially expressed miRNAs were performed using the R statistical language v. 3.1.2. The theoretical target genes of significantly expressed miRNAs were predicted by miRDB.

### Go biological process enrichment and KEGG pathway analyses

GO biological process enrichment and KEGG pathway of target genes of differentially expressed miRNAs were analyzed using the Molecule Annotation System (mas version 3.0, http://mas.capitalbiotech.online/mas3/). Genes are enriched significantly when p-value < 0.05 using a hypergeometric distribution. The q-value is the false discovery rate (FDR), wherein lower q-values indicate more significant enrichment of genes and less FDR.

### Statistical analysis

Data are represented as the mean ± standard deviation (SD) of three independent experiments. Statistical analyses were performed using the Statistical Package for the Social Sciences (SPSS version 16.0). Statistically significant differences were determined by the one-way ANOVA with a Fisher’s least significant difference (LSD) test. The values were considered significantly different at *p* < 0.05.

## Supplementary information

Supplementary Information

Dataset 1

Dataset 2

## Data Availability

The datasets generated during and analysed during the current study are available from the corresponding author on reasonable request.
