# Supplementary material for: Demethylation and microRNA differential expression regulate plasma-induced improvement of chicken sperm quality
Source: Sci Rep. 2019 Jun 20;9:8865. doi: 10.1038/s41598-019-45087-1 (PMC6586908; doi:10.1038/s41598-019-45087-1)
Supplement: Supplementary file 1 — Supplementary Information [file 41598_2019_45087_MOESM1_ESM.pdf]

Supplementary Information

Demethylation and microRNA differential expression regulate plasma-induced improvement of chicken sperm quality

Jiao Jiao Zhang[1], Nisansala Chandimali[2], Nameun Kim[2], Tae Yoon Kang[2], Seong Bong Kim[3], Ji Su Kim[4], Xian Zhong Wang[1,*], Taeho Kwon[4,*], Dong Kee Jeong[2,*]

[1] Chongqing Key Laboratory of Forage and Herbivore, College of Animal Science and Technology, Southwest University, Chongqing 400715, P. R. China

[2] Laboratory of Animal Genetic Engineering and Stem Cell Biology, Department of Advanced Convergence Technology and Science, Jeju National University, Jeju 63243, Republic of Korea

[3] Plasma Technology Research Center, National Fusion Research Institute, Gunsan-si, Jeollabuk-Do 54004, Republic of Korea

[4] Primate Resources Center, Korea Research Institute of Bioscience and Biotechnology, Jeongeup-si, Jeonbuk 56216, Republic of Korea

[*]Correspondence and requests for materials should be addressed to D.K.J. (E-mail: ngejeong@gmail.com), T.K. (E-mail: kwon@kribb.re.kr), and X.Z.W. (E-mail: xianzhong_wang@aliyun.com)

Supplementary Figures

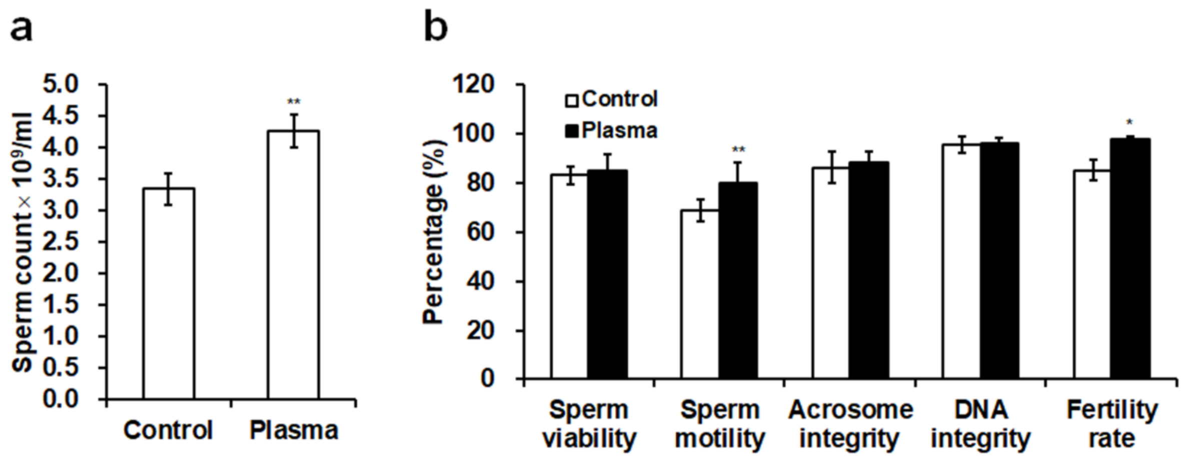

Supplementary Figure S1. Sperm quality of 20-week-old male chickens. (**a**) Sperm count. (**b**) Sperm viability, motility, integrities of acrosome and DNA, and fertility rate. Data are presented as the mean ± SD (n=10) of three replicates; n represents an individual chicken. *, $p < 0.05$ versus control; **, $p < 0.01$ versus control, according to the one-way ANOVA with a LSD test.

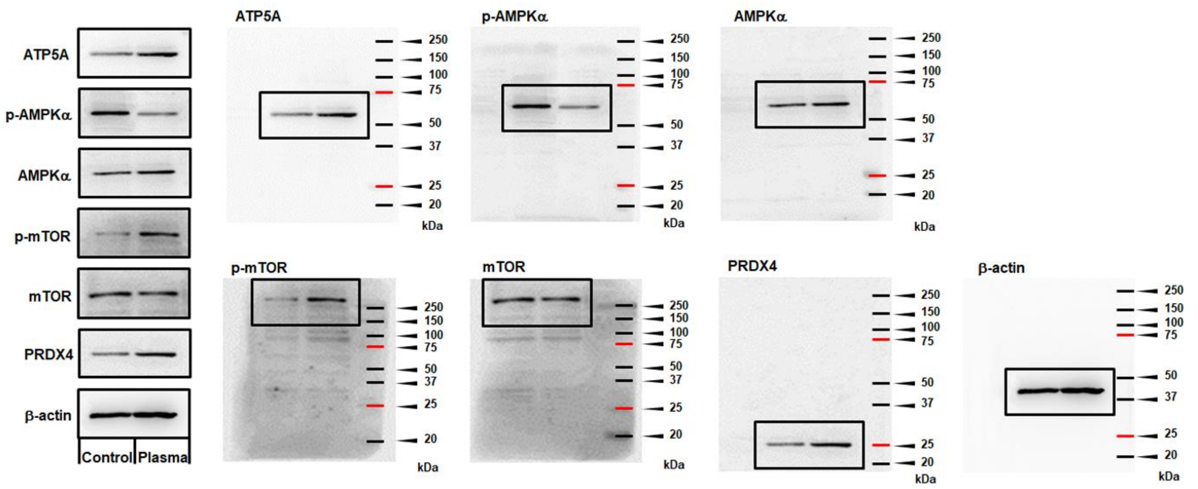

Supplementary Figure S2. Scans of all immunoblots for Figures 2d, 5g. The grouping of gels/blots cropped from different gels. All blots are visualized with 5 min exposure time.

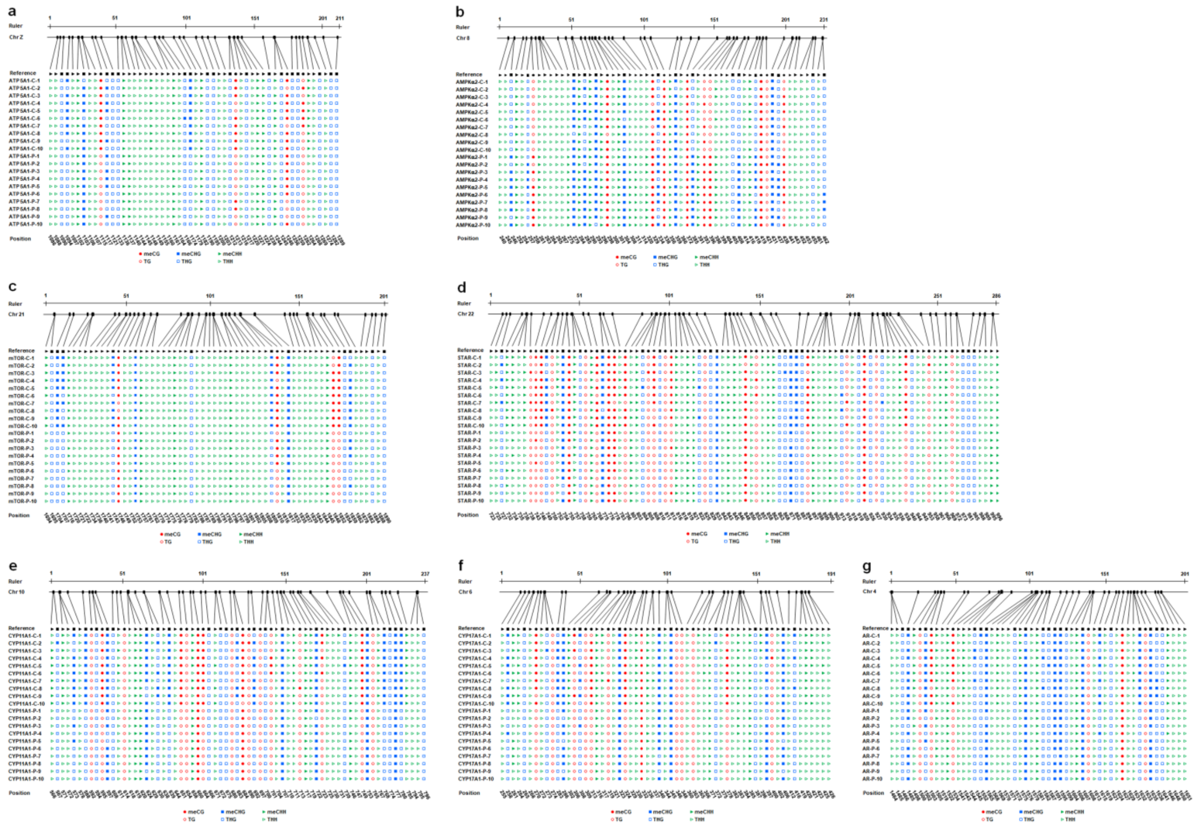

Supplementary Figure S3. Cytosine methylation analysis in 40-week-old male chickens. Bisulfite sequencing results of (**a**) *ATP5A1*, (**b**) *AMPK*α*2*, and (**c**) *mTOR* in the spermatozoa, (**d**) *STAR*, (**e**) *CYP11A1*, (**f**) *CYP17A1*, and (**g**) *AR* in the testis. The length of sequenced region and exact location of cytosine are shown schematically. The reference sequence is shown in the first line. The order of the individual sequences of ten clones is listed on the left. The sequence is distinguished by circles for CG, squares for CHG, and triangles for CHH. Filled symbols represent methylated cytosines, and open symbols represent unmethylated cytosines.

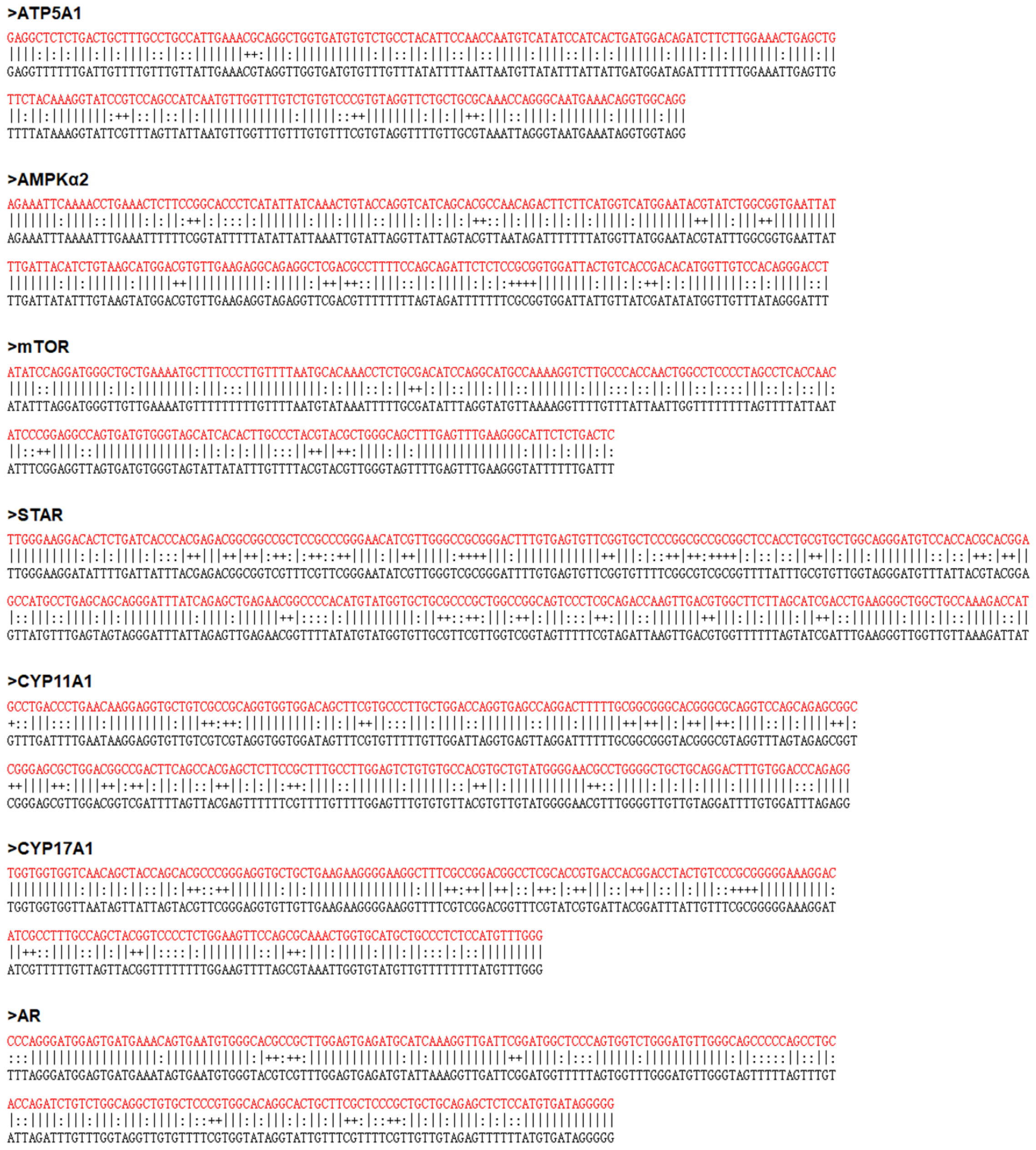

Supplementary Figure S4. The original sequences and the bisulfite converted sequences of sequenced region in *ATP5A1*, *AMPKα2*, *mTOR*, *STAR*, *CYP11A1*, *CYP17A1*, and *AR*. The original sequence is shown in upper row (red). The bisulfite converted sequence is shown in lower row (black). All CpG sites are assumed to be methylated for display. ++ represents CpG sites. :: represents non-CpG cytosine converted to thymine.

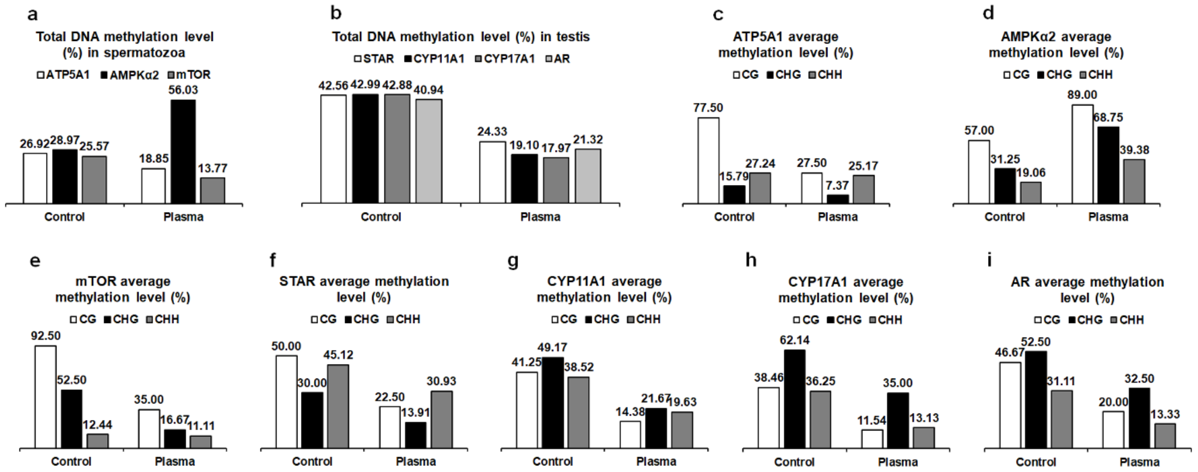

Supplementary Figure S5. One independent replicate on DNA methylation levels in the spermatozoa and testis of 40-week-old male chickens. Total DNA methylation levels in the sequenced regions of (**a**) *ATP5A1*, *AMPKα2*, and *mTOR* in the spermatozoa and (**b**) *STAR*, *CYP11A1*, *CYP17A1*, and *AR* in the testis. Average methylation levels for CG, CHG, and CHH in the sequenced regions of (**c**) *ATP5A1*, (**d**) *AMPKα2*, and (**e**) *mTOR* in the spermatozoa, (**f**) *STAR*, (**g**) *CYP11A1*, (**h**) *CYP17A1*, and (**i**) *AR* in the testis.

Supplementary Tables

Supplementary Table S3. KEGG pathway of target genes of differentially expressed miRNAs

Pathway	Count	p-value	q-value	Target gene of miRNAs
MAPK signaling pathway	15	1.44E-12	4.33E-12	*MAP3K2;MKNK2;RASA1;NF1;PTPRR;DUSP8;MAPK8;PPP3CA;RAPGEF2;NGFB;CACNA1E;RAP1A;FGFR3;SOS2;AKT3*
Wnt signaling pathway	8	7.83E-07	2.14E-07	*MAPK8;PPP3CA;RHOA;NKD1;SIAH1;CAMK2G;VANGL1;TBL1X*
Calcium signaling pathway	7	2.33E-05	2.91E-06	*PPP3CA;ATP2B1;CACNA1E;HTR4;ATP2A2;SLC25A4;CAMK2G*
ErbB signaling pathway	6	5.55E-06	1.01E-06	*GAB1;MAPK8;CBLB;SOS2;AKT3;CAMK2G*
Ubiquitin mediated proteolysis	6	6.42E-05	6.88E-06	*CBLB;MGRN1;RCJMB04_6c15;RCJMB04_7i20;SIAH1;UBE2O*
Apoptosis	5	4.77E-05	5.63E-06	*PPP3CA;NGFB;ENDOD1;CAPN1;AKT3*
p53 signaling pathway	5	1.76E-05	2.39E-06	*CCNE1;SESN1;EI24;SIAH1;PTEN*
mTOR signaling pathway	4	1.01E-04	9.78E-06	*ULK1;ULK2;EIF4E;AKT3*
Cell cycle	4	0.002507402	1.47E-04	*CCNE1;CDKN1B;RCJMB04_11l21;HDAC2*
Phosphatidylinositol signaling system	4	4.89E-04	3.76E-05	*PIP4K2A;PIK3C2A;PTEN;PIP5K1C*
Inositol phosphate metabolism	4	1.47E-04	1.38E-05	*PIP4K2A;PIK3C2A;PTEN;PIP5K1C*
Jak-STAT signaling pathway	3	0.028820349	0.001108475	*CBLB;SOS2;AKT3*
TGF-beta signaling pathway	3	0.00823066	4.05E-04	*RCJMB04_30p22;RHOA;PITX2*
Glycerophospholipid metabolism	3	0.002921604	1.69E-04	*PPAP2B;AGPAT3;ACHE*
Lysine degradation	3	0.001463448	8.78E-05	*WHSC1;PLOD2;SUV420H1*
Sphingolipid metabolism	3	9.55E-04	6.23E-05	*PPAP2B;SGMS1;SMPD3*
Nucleotide excision repair	3	8.69E-04	5.98E-05	*ERCC4;RAD23B;GTF2H1*
Ether lipid metabolism	3	4.57E-04	3.61E-05	*PPAP2B;AGPAT3;PAFAH1B1*
Glycosphingolipid biosynthesis - lactoseries	3	1.67E-04	1.52E-05	*ST3GAL6;B3GNT2;B4GALT2*
Keratan sulfate biosynthesis	3	5.99E-05	6.66E-06	*CHST1;B3GNT2;B4GALT2*
VEGF signaling pathway	2	0.044025732	0.001492398	*PPP3CA;AKT3*
Notch signaling pathway	2	0.026858875	0.00104645	*HDAC2;RBPJ*
N-Glycan biosynthesis	2	0.020122244	8.27E-04	*ALG5;B4GALT2*

Valine, leucine and isoleucine degradation	2	0.019079385	7.95E-04	*BCAT1;MUT*
Glycerolipid metabolism	2	0.018060204	7.64E-04	*PPAP2B;AGPAT3*
SNARE interactions in vesicular transport	2	0.015147991	6.59E-04	*STX16;SNAP23*
Glutathione metabolism	2	0.014226797	6.32E-04	*SRM;GCLM*
Retinol metabolism	2	0.010798736	5.06E-04	*RDH5;RDH12*
Regulation of autophagy	2	0.005247652	2.74E-04	*ULK1;ULK2*
Alkaloid biosynthesis II	1	0.041684461	0.001479922	*RCJMB04_35i21*
Valine, leucine and isoleucine biosynthesis	1	0.041684461	0.001479922	*BCAT1*
Terpenoid biosynthesis	1	0.018081078	7.64E-04	*FDFT1*

KEGG pathway of target genes of differentially expressed miRNAs in male chicken testis is analyzed using Molecule Annotation System version 3.0. Target genes are enriched significantly in the pathway when p-value < 0.05 using a hypergeometric distribution. The q-value is the FDR, wherein lower q-values indicate more significant enrichment of target genes and less FDR.

Supplementary Table S4. Chicken sperm RNA quality and concentration

**Group**	**Absorbance at 260/280**	**RNA concentration (ng/µl)**
Control	1.94	198.2
Plasma	2.00	176.6

Supplementary Table S5. Primer sequences for RT-PCR

Gene	Sequence number	Sequence position	Product length (bp)	Annealing temperature (℃)	Sequence (5'to3')
β-actin	NM_205518.1	625-818	194	57	F: GTGCGTGACATCAAGGAGAAGC<br>R: CCACAGGACTCCATACCCAAGA
ATP5A1	NM_204286.1	1207-1364	158	57	F: GGTATCCGTCCAGCCATCAA<br>R: GCATCCAAATCAGACCCAAACT
ATP5B	NM_001031391.2	482-637	156	57	F: GCCCCATCACAACGAAACAG<br>R: CGCCTCCAAACAAACCAATC
ATP5C1	NM_001278096.1	272-411	140	57	F: ATTAAGGCACCCGAGGACAA<br>R: ACTTCCTTCCCTGCATTGGA
ATP5F1	XM_417993.4	437-644	208	57	F: CATTGGAGACTGCCATTGAGG<br>R: TGATCTTGCTCTTTCTGACGCTT
ATP5G1	XM_001233602.3	287-536	250	57	F: CAGGAGCAGGTATTGGGACA<br>R: TTGTCAGTCTGGAACGCTCT
ATP5G3	NM_001277855.1	141-288	148	57	F: CCAAAACGCTGTCTCCCAAC<br>R: ACCGAAGACCGTTCCAATACC
ATP5H	XM_001232598.3	332-551	220	57	F: CTGAAGGTCCCTGAACCAGT<br>R: ACTTCTCCCTGTCCAGTCTG
ATP5I	NM_001097534.2	74-240	167	57	F: TCTCGCCCCTCATCAAGTTC<br>R: TGCCAGTTCCTTTGCAATCC
ATP5J	XM_004938370.1	58-197	140	58	F: CACTTGCGGAGAAACATCGGT<br>R: CCTACATCAACAGGTCCTCCAGC
ATP5J2	NM_001257200.1	170-263	94	57	F: GCCTCGGTGGTATCAGTATGGT<br>R: TACTTCCTGCGGCGGTCAT
ATP5L	XM_015298211	250-377	128	57	F: CCATGGTCAGGAGCTTTCAG<br>R: GCCTCGTTTGCCTATGATCTC
ATP5S	NM_001277562.1	46-279	234	57	F: TCCCCTTCCCCTTTCTTTCC<br>R: CATAGCCTTGATAGCGCACC
AMPKα2	NM_001039605.1	726-943	218	57	F: GGAGGCGTGTTTTACATCCC<br>R: AACTTCTCACAGACCTCCCG
AMPKβ2	NM_001044662.1	435-661	227	57	F: CCAGTGTTTTCAGCTCCCAC<br>R: GAGGTCCAGGATAGCGACAA
AMPKγ3	NM_001031258.2	183-320	138	57	F: GCTGGAACCCGACAACAATT<br>R: GCCTTCTTGATCTCCAGGGT
mTOR	XM_417614.4	119-309	191	57	F: TGAAGGGGTCAAGGCAATCC<br>R: GGCGAGCAGTGGTTGTGGAT
PRDX1	NM_001271932.1	358-545	188	56	F: ACAAGGTGGTTTGGGCACTA<br>R: TCTCATCAACAGAACGGCCA
PRDX3	XM_426543.5	414-551	138	56	F: TTTCACCTTTGTGTGCCCCA<br>R: TTGCGCGGGGTATTTATCCA

PRDX4	XM_001233999.3	595-733	139	56	F: TGCACTTAGGGGCCTTTTCA<br>R: TTCTCCATGCTTGTCCGTGT
PRDX6	NM_001039329.2	189-340	152	58	F: TGAGTTCAGCAAACGCAACG<br>R: GCTCTCGGTCCTTATCAGCG
STAR	NM_204686.2	543-726	184	57	F: CAACGGAGACAAAGTGCTGA<br>R: AGTGTCCTTCCCAACCCTCT
CYP11A1	NM_001001756.1	1013-1121	109	57	F: CATCCTCTTCAGCCTCCTTG<br>R: TTGCAGAGTCATGGAAGTCG
CYP17A1	NM_001001901.2	673-804	132	58	F: GACATCTTCCCCTGGCTACA<br>R: CACAGTGTCCCCACAGAATG
HSD17B3	XM_425046.5	916-1064	149	57	F: ACTCCAATGACAATGCACCA<br>R: GGAACAAGCTGCAGAACACA
AR	NM_001040090.1	1615-1843	229	58	F: CTGGGCAGTCTGAAGACACA<br>R: TCTCCCCAAGTTCATTCAGG

Supplementary Table S6. Primer sequences for bisulfite-sequencing PCR

Gene	Sequence position	Product length (bp)	Annealing Temperature (℃)	Sequence (5'to3')	Expected No. of CpGs
ATP 5A1	1082-1292	211	55	F: GAGGTTTTTTGATTGTTTTGTTTGT<br>R: CCTACCACCTATTTCATTACCCTAAT	4
AMPKα2	233-463	231	52	F: AGAAATTTAAAATTTGAAATTTTTT<br>R: AAATCCCTATAAACAACCATATATC	10
mTOR	1690-1890	201	52	F: ATATTTAGGATGGGTTGTTGAAAAT<br>R: AAATCAAAAAATACCCTTCAAACTC	4
STAR	713-998	286	52	F: TTGGGAAGGATATTTTGATTATTTA<br>R: ATAATCTTTAACAACCAACCCTTCA	24
CYP11A1	565-801	237	53	F: GTTTGATTTTGAATAAGGAGGTGTT<br>R: CCTCTAAATCCACAAAATCCTACAA	16
CYP17A1	245-435	191	51	F: TGGTGGTGGTTAATAGTTATTAGTA<br>R: CCCAAACATAAAAAAAACAACATAC	13
AR	1464-1664	201	53	F: TTTAGGGATGGAGTGATGAAATAGT<br>R: CCCCCTATCACATAAAAAACTCTAC	6

Supplementary Table S7. Primary and secondary antibodies used in study and their respective dilutions

	**Target**	**Name of antibody**	**Source and reference**	**Species raised in; clonality**	**Dilution used**
Primary antibodies	p-AMPKα	AMPK alpha1 + AMPK alpha2 (phospho T172)	Abcam, Cambridge, UK	Rabbit; polyclonal	1:1,000
	AMPKα	Anti-AMPK alpha1/AMPK alpha2	Abcam	Mouse; monoclonal	1:1,000
	p-mTOR	mTOR (phospho S2448)	Abcam	Rabbit; polyclonal	1:1,000
	mTOR	Anti-mTOR	Abcam	Rabbit; polyclonal	1:1,000
	ATP5A	Anti-ATP5A	Abcam	Mouse; monoclonal	1:250
	PRDX4	Peroxiredoxin 4	LifeSpan BioSciences, Seattle, WA, USA	Mouse; monoclonal	1:200
	Beta-actin	Anti-beta actin	Santa Cruz Biotechnology, Dallas, Texas, USA	Rabbit; polyclonal	1:1,000
Secondary antibodies	Goat IgG	Anti-rabbit IgG H&L (HRP)	Abcam	Goat; polyclonal	1: 5,000
	Goat IgG	Anti-mouse IgG-HRP	Santa Cruz Biotechnology	Goat; polyclonal	1: 5,000
